# Qualitative Research About Attributions, Narratives, and Support for Obesity Policy, 2008

**Published:** 2011-02-15

**Authors:** Jeff Niederdeppe, Stephanie A. Robert, David A. Kindig

**Affiliations:** Cornell University; University of Wisconsin-Madison, Madison, Wisconsin; University of Wisconsin-Madison, Madison, Wisconsin

## Abstract

**Introduction:**

Successful efforts to reduce obesity will require public policy strategies that target both individuals and external factors such as social conditions, economic circumstances, and physical environments. Public opinion data suggest that many policy changes to reduce obesity are likely to face public resistance.

**Methods:**

We conducted 4 focus groups involving 33 adults living in or near a midsized Midwestern city in July 2008. Participants were assigned to the focus groups on the basis of self-reported political ideology. We used a semistructured discussion guide to 1) better understand public perceptions of obesity and 2) assess the promise of narratives as a strategy to stimulate meaningful discussion about obesity-related policy change.

**Results:**

Participants viewed internal factors as primary causes of obesity. Despite substantial acknowledgment of external causes of obesity, many participants — particularly political conservatives — were resistant to external policy solutions for the problem. Across the political spectrum, participants responded more favorably to a short narrative emphasizing barriers to reducing adult obesity than a story emphasizing barriers to reducing childhood obesity.

**Conclusion:**

This study provides a deeper context for understanding public perceptions about obesity. Some types of narratives appear promising for promoting support for policy solutions to reduce obesity.

## Introduction

Obesity rates are rapidly increasing in the United States, resulting in greater chronic disease risk and reduced quality of life (1). Successful obesity reduction efforts will require new policies to modify external factors such as social, economic, and physical environments (2,3). Policy change to reduce obesity is unlikely without public support. Opinion polls show only mixed support for obesity-reducing policies such as subsidies for fruits and vegetables or zoning laws that promote physical activity and healthy food availability (4,5). More effective communication strategies are needed to increase awareness of social determinants of obesity and promote support for policy changes that target these determinants.

Literature on attributions and narratives (personal stories) can help inform these strategies (6). Attribution theory says that people attribute the causes of other people's dispositions as within a person's control (internal) (eg, too lazy to exercise) or outside of a person's control (external) (eg, lack of safe places for exercise) (7,8). People who think obesity is within internal control are less likely to support policies to create healthier social, economic, and physical (external) environments (9,10). Politically, conservatives are more likely than liberals to think obesity is within internal control (9), less likely to support obesity policies (10), and less responsive to news stories highlighting social determinants of obesity (11).

Narratives facilitate attitude and behavior change by connecting readers with characters that represent broader populations (12,13). Personal stories can successfully emphasize structural causes of social problems (14,15) and are a part of many recent campaigns to increase awareness of social determinants of health (16). We used focus group data to explore issues involved with the development of narratives to promote policies for reducing obesity rates. We asked 2 research questions: How do participants conceptualize attributions for obesity in their own terms? How do attributions and responses to obesity narratives vary by political partisanship or topic (eg, adult vs childhood obesity)?

## Methods

### Design and setting

Thirty-three adults from the metropolitan area of a midsized Midwestern city participated in 1 of 4 focus groups in July 2008. We used a sample of listed telephone numbers to recruit adult participants. Inclusion criteria required participants to intend to vote in the 2008 presidential election and to self-identify as a Democrat, Republican, liberal Independent/Other Party, or conservative Independent/Other Party. We excluded Independents and members of other parties if they self-identified as politically moderate. We asked eligible participants to participate in a focus group about health and weight-related issues. To avoid polarized discussions about attributions and policy (9-11), we assigned participants to groups by political ideology, producing 2 sessions for liberals/Democrats, Lib A and Lib B, and 2 sessions for conservatives/Republicans, Cons A and Cons B.

### Participants

Participants' ages ranged from 30 to 80 (mean, 54 y); 15 were women, and most (n = 30) were white. Seventeen had a college degree, and 14 had attended some college. More than half (n = 21) of participants had no children. Fourteen were married, 10 divorced, 6 never married, and 3 widowed. Ten participants were obese (body mass index [BMI] ≥30.0 kg/m^2^) and 11 were overweight (BMI ≥25.0 kg/m^2 ^and <30.0 kg/m^2^; overall BMI mean, 27.8 kg/m^2^; and standard deviation, 5.7).

### Focus group protocol

The University of Wisconsin's Social and Behavioral Sciences institutional review board approved the study. A trained moderator led participants in 2-hour focus group sessions, using the same base questions and probes about factors that cause obesity, solutions for high rates of obesity, and attributions of responsibility for solving the problem (Appendix). Halfway through the session, we asked participants to read 1 of 2 narratives emphasizing external causes of obesity and to share their thoughts on the story. After the discussion, participants completed a short demographic questionnaire. Two groups read a narrative that framed obesity as an adult issue (www.cdc.gov/obesity) and 2 groups read a narrative that framed obesity as a childhood issue (www.rwjf.org/childhoodobesity).

### Narratives

**Adult obesity narrative**. One group from each political ideology (Lib A and Cons A) read a story about a young adult named John Stevenson who lived in a poor neighborhood and had difficulty losing weight. We adapted and made several modifications to the story of a real person depicted by the Robert Wood Johnson Foundation's (16) Commission to Build a Healthier America. We changed the race of the main character from black to white to avoid priming racial stereotypes (17). The story acknowledged personal decisions (internal causes) but emphasized social barriers, including cost and availability of healthy food, stress associated with a low-income job, and lack of safe and affordable places for exercise (external causes).

**Childhood obesity narrative.** The other groups (Lib B and Cons B) read a story about a boy named Jimmy Collins who lived in a rural area and had steadily gained weight during the past year. We adapted the story of a real family depicted in a *Washington Post* article and supplemented it with material from a *Time *article (18,19). We changed the race of the family from Hispanic to white to avoid priming racial stereotypes. The story described weight-loss strategies that Jimmy and his mother tried (internal causes); it also described time, financial, and availability barriers faced by Jimmy and his parents (external causes).

### Data analysis

The first author used N6 qualitative analysis software (QSR International, Cambridge, Massachusetts) to identify recurring themes and to classify statements in transcripts of each discussion. Coding began with a set of categories that mirrored the structure of the focus group protocol but also allowed for the inductive identification of codes as new themes emerged. On the basis of an ecological framework, we classified causes of obesity as internal or external (2). We classified solutions to obesity into 4 categories: 1) internal — alone, 2) internal — educational, 3) external — social/organizational, and 4) external — legislative. Although a focus on individual knowledge through education implies external involvement, we classified this strategy as internal because it ultimately places responsibility on individuals to acquire knowledge and put it into practice (1,20,21). Furthermore, although receiving social support or participating in school or workplace initiatives often requires some individual motivation, we classified these strategies as external because they require effort or investment from people or organizations beyond the individual. We highlight differences in response by ideology (liberal vs conservative) or exposure to the 2 narratives (adult vs childhood) only when clear patterns of difference emerged across groups.

## Results

### Perceived causes of obesity: prenarrative exposure

**Internal causes.** All but 1 group began the discussion about causes of obesity by focusing on diet and exercise. Regardless of political ideology, participants most frequently identified 3 internal causal attributions: 1) intrinsic individual dispositions (eg, lazy, unmotivated); 2) lack of knowledge and skills; and 3) genetic, medical, or biological causes. For instance, 1 woman (Lib A) described intrinsic individual dispositions: "lazy, eating so much . . . and not getting enough exercise, and not doing anything with their body."

Across groups, several participants noted the challenge of making good dietary choices amid uncertain scientific recommendations and limited knowledge. Several expressed sympathy for mental or physical health problems. Still, most participants who recognized these constraints conveyed a dominant ideology of internal responsibility. One quote illustrates this view:

Some people are overweight regardless of how much exercise they do, or if they eat lettuce and carrots all day . . . and there's very little they can do about it. However, I know also that there is a very strong correlation between eating junk food and watching a lot of television. So when I see kind of a chubby person walking by, my gut feeling is, 'Oh, there goes a lazy person.' . . . That really isn't a fair assessment, but . . . that's the reality of it. (woman, Lib A)

Overall, liberal and conservative groups conveyed strong beliefs that individual decisions about diet and exercise play a large role in causing obesity.

**External causes.** Each group also identified external causes of obesity, including 1) food availability and price; 2) family composition and time; 3) institutional culture and policy; and 4) characteristics of the physical or media environment. These themes emerged with no clear patterns of difference between liberals and conservatives.

Many participants described how, unlike healthy foods, unhealthy foods were prevalent and affordable. Portion size and time constraints related to family commitments also emerged as recurrent themes. One male participant (Cons A) commented, "Back when I grew up, my dad worked and my mom stayed home and cooked the meals. That doesn't happen anymore. . . . Everybody's working 2 jobs just to survive, so there's not a lot of time for staying home and making meals."

Many cited time constraints from long and stressful work hours as causes of obesity. Some discussed workplace culture, which may encourage unhealthy food decisions, or mentioned the lack of physical and nutrition education and the availability of junk food in schools.

Each group identified the physical and media environments as causes of overweight. Participants mentioned extreme weather, neighborhood safety, city sprawl, lack of public transport, and other elements of city design as impediments to exercise. Several participants made explicit comparisons between the United States and other countries: "Most of Europe did not evolve with the automobile the way we did and [Europe] . . . is set up in such a way that it is easy to walk and bicycle a lot of places" (woman, Lib A). Participants in each group also discussed ways that television, video games, and other media encourage sedentary behavior (time not spent exercising) and unhealthy diets (aggressive marketing of unhealthy food).

### Perceived solutions to high rates of obesity: prenarrative exposure

**Internal — alone.** Although each group offered several possible solutions to high rates of obesity, many participants voiced the opinion that responsibility for obesity rests solely with individuals (internal) or their parents (if they are children). These sentiments were offered more frequently in conservative groups but also appeared regularly among liberals.

**Internal — educational.** The internally focused strategies were the most common solutions cited among both liberals and conservatives: 1) strengthening individual knowledge, particularly in schools, and 2) community education such as public education campaigns at the community or national level. These strategies imply some degree of public investment but ultimately reflect internal attributions: people are responsible for making good decisions about diet and exercise when information is available. Before reading the narrative, each group concluded that enhancing diet and exercise-related education was the best strategy for reducing obesity rates.

**External.** Participants offered 3 types of external solutions, although less frequently than internal solutions: 1) increasing social support (without legislation), 2) changing organizational practices (without legislation), and 3) influencing public policy and legislation. Liberals and conservatives each discussed social support and organizational practices, but clear ideological differences emerged for legislation to help reduce obesity rates.

**External — social/organizational.** Three of the 4 groups mentioned the importance of increasing social support for nutrition and exercise. Each group also discussed the importance of organizations, including schools (lunch programs, vending machines, recess, and exercise facilities), insurance companies (incentives for diet and exercise), workplaces (onsite facilities and classes), restaurants (healthier choices and reduced portion size), or businesses (target fitness clubs or food delivery services to overweight, elderly, or low-income populations).

**External — legislative.** Liberal groups offered a few legislative options to reduce obesity. Liberal groups discussed junk food taxes to reduce unhealthy food consumption and gasoline taxes to encourage physical activity. Several conservative participants explicitly rejected these ideas without prompt. For instance, 1 man (Cons B) said, "Boy, not taxes. Make sure that's in there [laughter, agreement]." Liberal groups also discussed food advertising bans, mandatory media-use restrictions, and city planning for healthier environments. Conservative groups mentioned only mandatory food labeling as a public policy. Although legislative, this strategy implies internal responsibility for making informed decisions about diet.

### Resonant themes in response to obesity narratives

We observed striking differences in the responses to the adult and childhood obesity narratives. These differences stood in stark contrast to the minor differences observed between liberal and conservative groups.

**Adult obesity narrative.** A common response to the adult story focused on factors external to John, such as the social, economic, and physical conditions that contribute to obesity. For instance, 1 man (Cons A) said, "What stresses people out [is] when they don't have enough money and they . . . make poor decisions. . . . Well this is cheaper, may not be as good for me, but it will fill me up." Another woman (Lib A) summarized, "It seems like from reading . . . that society has stacked a lot of things against him."

Empathy and identification with John's intrinsic, internal characteristics reflected a second prominent theme in response to the adult narrative. As 1 man (Cons A) commented, "I saw me in there. . . . I saw a lot of similarities to my lifestyles or my family and friends, stuff like . . . stress, cost, convenience, good choices." Overall, participants who read the adult narrative empathized with the barriers John faced in reducing his weight and admired his resolve in trying to overcome them.

**Childhood obesity narrative.** In contrast, comments about the childhood story focused on internal attributions and decisions made by Jimmy's parents. Some of these criticisms were directly related to Jimmy's mother's attempts at managing Jimmy's weight. One man commented (Lib B), "It seems like she's spending money all the time and just throwing it away." Several participants said Jimmy's parents were "making excuses" or criticized their level of involvement with him. Still others focused on incidental aspects of the story that were viewed as broader indicators of bad parenting: "That little kid is allowed to play Halo [a first-person shooter video game], and it's not, not a good parent to let him play Halo" (man, Cons B).

Narrative exposure prompted substantial discussion about possible solutions to childhood obesity. However, across political ideologies, most discussions ultimately placed most of the responsibility in the hands of parents, often commenting on the need for obesity education. These statements acknowledge that parents need more information but focus on internal parental decisions as paramount for reducing obesity in children.

### Most important solution: postnarrative exposure

After discussing reactions to the stories, we asked participants to identify the single most important thing that could be done to reduce obesity rates. More than half of the participants that read the adult story responded with external solutions (eg, insurance company incentives, workplace programs, tax policies). In contrast, only 1 participant in each of the 2 groups that read the childhood story mentioned external solutions (eg, universal health care, restaurant portion size restrictions).

## Discussion

The analysis revealed 4 main themes. First, although both liberal and conservative participants viewed internal factors as the primary causes of obesity, most also acknowledged that external factors play an important role. Second, regardless of political ideology, participants offered internal solutions focused on reducing knowledge deficiencies as primary strategies to reduce rates of obesity. Third, consistent with previous work (10), conservatives voiced resistance to many external solutions, particularly legislative options. Fourth, both liberals and conservatives responded more favorably to a story emphasizing barriers to reducing adult obesity than to one emphasizing barriers to reducing childhood obesity, despite comparable efforts by the characters to reduce their (or their child's) weight.

Consistent with previous work, our findings suggest that many adults conceptualize obesity as a condition that is primarily the responsibility of individuals or parents (9). Internal responsibility was the starting point for discussions about external causes, and many participants rejected the idea of legislative solutions to address obesity. Nevertheless, obesity-reducing efforts will be unsuccessful without policy changes. These factors underscore the importance of developing messages that acknowledge individual decisions while emphasizing external causes and solutions. Future studies might assess, develop, and test strategies to 1) acknowledge individual decisions but 2) refute the idea that these factors *alone* cause obesity (11,22).

Both liberals and conservatives identified several external causes of obesity. Nevertheless, participants in all groups offered few external solutions to address these issues. These results suggest that advocates and researchers would benefit by developing and testing strategies to raise public awareness of other policy and legislative options at multiple levels of intervention (eg, school, workplace, community, national).

Findings also suggest that some types of narratives may promote support for policy solutions. Both liberal and conservative groups expressed empathy for the adult story's main character and focused their attention on external factors that cause obesity. Narratives that generate empathy and call attention to external causes of obesity thus appear to have the potential to generate support for obesity-related policy change (8). At the same time, little is known about specific elements of narratives that maximize effectiveness in behavior or policy change (13).

The childhood obesity narrative, although explicitly designed to emphasize external causes, generated substantial criticism toward the child's parents. A focus on childhood obesity may inadvertently prime or activate existing beliefs about parental attribution of responsibility for childhood obesity (23,24). Future work should assess whether different narratives are more effective in promoting broader societal concern and attribution of responsibility for childhood obesity.

### Limitations

This study reports on 4 discussions among a small sample of predominately white adults living in a midsized Midwestern community. Results are not generalizable to the broader US population but are meant to serve as a starting point for further empirical testing of effective message strategies for upstream policy change. Although both messages were designed to emphasize external barriers to achieving a healthy weight, there are other ways to tell stories that emphasize these issues. The 2 stories differed substantially in plot (losing weight vs avoiding gaining weight), character (male [John] vs female [Jimmy's mom]), and setting (urban vs rural). Differences in these factors may also explain differences in audience responses to them. In addition, the demographic composition of the sample (ie, predominately white and more than half without children) may have shaped responses to the adult and child obesity narratives.

### Conclusion

This study's results provide a deeper context of understanding public perceptions about obesity. Results suggest that some narratives emphasizing barriers to diet and exercise may help to generate support for obesity-related policy change. Next steps include 1) formally testing the effectiveness of narrative message strategies for generating support for specific policies and 2) identifying specific story elements that enhance or reduce effectiveness.

## Appendix. List and Order of Questions That Were Asked Within Each Focus Group, 2008

1. Let's start out by talking about health. There are a lot of different aspects of a person's health. In your view, what does it mean to be healthy?
2. Now, let's talk about a specific health issue that many people are talking about these days — the number of ________ residents who are overweight. When you hear the word "overweight," what different kinds of things come to mind?
3. How about the word "obese?" What different kinds of things come to mind when you hear this word?
4. What factors cause someone to be overweight?
  - PROBE (If person mentions diet or exercise): Why do you think that some people don't (eat healthy foods or exercise) as often as they should?
5. When you are making decisions about the kinds of foods you eat, what sorts of things influence your decisions?
6. Now let's talk a little bit about exercise. When you are making decisions about whether or not to exercise, what sorts of things influence your decisions?
7. What kinds of challenges or barriers have you personally faced in trying to eat healthy foods or exercise regularly?
  - PROBE: Can you describe to me any situations you have been in where it was very difficult to eat healthy foods or exercise?
8. Are you concerned about the number of ________ residents who are overweight?
  - PROBE: How much of a concern is this issue to you?
  - PROBE: (If they are not concerned) Why not?
  - PROBE: (If they are concerned) What about it makes you concerned?
9. Do you think that certain groups of people tend to be more overweight than others in this country?
  - PROBE: In your opinion, which groups would this be?
  - PROBE: Why do you think these differences exist?
10. We talked tonight about many different causes of weight-related health issues. Can you think of any possible solutions to helping reduce the number of ________ residents who are overweight?
  - PROBE: Which sorts of things would reduce the number of ________ residents who are overweight?
11. Whose responsibility is it to do something to reduce the number of ________ residents who are overweight?
  - PROBE: Is there a way that society can help individuals eat right and exercise more?
  Show message 1, read aloud, and provide 3-4 minutes for notes. As you are reading this message, I want you to write down on a piece of paper everything that comes to your mind as you are reading it. Don't write out complete sentences; just write your initial thoughts as you go through the story.
12. What kind of thoughts came to your mind as you were reading this story?
  - PROBE: At what point in the story did you think this?
13. What message do you think this story sends?
14. Did you find this story believable?
  - PROBE: How much are you able to identify with the character?
  - PROBE: In what ways?
15. What parts of this story did you find the most compelling or realistic?
16. Were there any parts of this story that were unrealistic or didn't make sense to you?
17. What role, if any, do you think society has to play in helping the character in the story (eat healthier or exercise more)?
18. We talked about many different issues tonight related to health and weight. Considering all of the factors that affect weight-related issues and health, which ONE factor do you consider to be most important in reducing the number of ________ residents who are overweight?
19. Finally, is there anything related to health and weight-related issues that we didn't talk about tonight that you'd like to have the opportunity to share?
